# Tailoring Thermal Transport Properties of Graphene Paper by Structural Engineering

**DOI:** 10.1038/s41598-018-38106-0

**Published:** 2019-03-14

**Authors:** Li Ren, Mengjie Wang, Shaorong Lu, Lulu Pan, Zhongqiang Xiong, Zuocai Zhang, Qingyuan Peng, Yuqi Li, Jinhong Yu

**Affiliations:** 1 Key Laboratory of New Processing Technology for Nonferrous Metals and Materials, Ministry of Education, School of Material Science and Engineering, Guilin University of Technology, Guilin, 541004 China; 2 Key Laboratory of Marine Materials and Relater Technologies, Zhejiang Key Laboratory of Marine Materials and Protective Technologies, Ningbo Institute of Materials Technology and Engineering, Chinese Academy of Sciences, Ningbo, 315201 China

## Abstract

As a two-dimensional material, graphene has attracted increasing attention as heat dissipation material owing to its excellent thermal transport property. In this work, we fabricated sisal nanocrystalline cellulose/functionalized graphene papers (NPGs) with high thermal conductivity by vacuum-assisted self-assembly method. The papers exhibit in-plane thermal conductivity as high as 21.05 W m^−1^ K^−1^ with a thermal conductivity enhancement of 403% from the pure cellulose paper. The good thermal transport properties of NPGs are attributed to the strong hydrogen-bonding interaction between nanocrystalline cellulose and functionalized graphene and the well alignment structure of NPGs.

## Introduction

Nowadays, solving the heat accumulation problem is a top priority for obtaining good-performance and long-lifetime flexible electronic devices, which have become more miniaturized, integrated and functional^1^. Studying high thermal conductivity (TC) of polymeric composites has been considered an effective way to solve this problem^2^. Graphene, a sp^2^-bond two-dimensional material, has a super thermal conductivity (~5300 W m^−1^ K^−1^), whose conjugated molecular structure provides an ideal two-dimensional channel for phonon propagation^3,4^. Besides, micron-grade graphene has a large contact area with the polymer matrix compared to the zero- or one-dimensional fillers, such as fullerene^5^, ceramics^6,7^, metal nanoparticles^8,9^, carbon nanofiber^10^ and so on. So graphene is regarded as a kind of favor fillers for high TC for polymer-based composites. For example, Shen *et al*. employed multilayer graphene as fillers associated with epoxy to fabricate thermal conductive composites. It was found that the obtained composites at 10 vol% graphene loading showed the TC of 5.1 W m^−1^ K^−1^ ^11^. Li *et al*. fabricated nanofibrillated cellulose/graphite nanoplatelets (GNPs) composite papers reached a TC of 12.4 and 27.95 W m^−1^ K^−1^ at 10 wt% and 25 wt% GNPs loading, respectively^12^. Though the higher TC values will be enhanced with the increase of the graphene content, the enhancement of the TC grows slowly due to the poor dispersion of graphene in the polymer matrix. So how to improve the dispersion of graphene in polymer is still a challenging problem.

It was found that graphene functionalization (either covalent or noncovalent functionalization) is a common and efficient method to enhance a good dispersion of graphene in polymers^13–16^. Generally, covalent functionalization introduces defects or distortions into the graphene basal plane during the synthesis of graphene oxide, compromising the sp^2^ structure of graphene lattices and leading to a loss of the electronic properties. In contrast, noncovalent functionalization, such as *π*–*π* stacking interaction is a better option because it does not alter the structure or electronic properties of the graphene while it simultaneously introduces new functional groups on the surface^15,17^. Teng *et al*. adopted functional segmented poly(glycidyl methadrylate) containing localized pyrene groups to functionalize graphene nanosheets (GNSs) through *π*–*π* stacking interaction and obtained a homogeneous dispersion liquid (Py-PGMA-GNS). The Py-PGMA-GNS was then dispersed in an epoxy matrix to fabricate Py-PGMA-GNS/epoxy composites. The TC of Py-PGMA-GNS/epoxy with only 4 phr loading achieved 1.19 W m^−1^ K^−1^ and was 20% higher than that of the composites with pristine graphene^18^. What’s more, different functional molecules including 1-pyrenebutyl, 1-pyrenebutyric and 1-pyrenebutylamine can reduce the interfacial thermal resistance by noncovalent functionalization reported by Wang *et al*.^19^. It provided a theoretical basis for improving thermal conductivity of polymer composites because the low thermal interface resistance can lead to an enhancement of thermal conductivity. Thus surface noncovalent functionalization for fillers is an efficient way to enhance the interaction between the fillers and the polymer matrix^20,21^.

Perylene-3, 4, 9, 10-tetracarboxylic acid dimide derivatives (PBI) have been extensively used in organic semiconductors, photovaltaic devices and fluorescence spectroscopy^22^. PBIs have a large electron-rich planar aromatic structures that is benefit for their strongly anchor onto the surface of graphene sheets via *π*–*π* stacking interaction^23^. Up to now, there were many reports related to the perylene dimides functionalized graphene and improved the dispersion of graphene^24–27^. In a recent report, a series of ionic formed polyamine-functionalized PBIs (HAPBI) were synthesized to disperse graphene via *π*–*π* stacking interaction, a high concentration of 1.0 mg ml^−1^ graphene dispersion was obtained at the 1:3 weight ratio of HAPBI to graphene^27^.

Cellulose is a renewable and sustainable eco-friendly natural material. The nanocellulose-based thermal conductive nanocomposites have attracted much attention, which exhibited remarkable mechanical strength, anisotropy and flexibility due to its highly ordered hierarchical structures^28–31^. Nanocellulose includes cellulose nanofibers (CNF) and nanocrystalline cellulose (NCC). Particularly, NCC, rod-like nanoparticles (5–20 nm wide and 50–500 nm long), was conventionally prepared by acid hydrolysis, which introduces many hydrophilic groups on the surface of NCC^32–34^.

In this work, NCC was extracted from sisal fibers by three main steps, de-waxing process, bleaching process, grafting process and a series of mechanical process. Then high thermal conductive sisal nanocrystalline cellulose/noncovalent functionalized graphene composite papers (NPGs) were prepared by vacuum-assisted self-assembly method. A polyamine-functionalized PBI (PED) was synthesized and further used as the stabilizer of graphene. It was found that the high concentration of the stable graphene dispersion can reach up to 0.5 mg ml^−1^ using the PED as a stabilizer. Meanwhile, the PEG and NCC matrix can form stable dispersions due to the hydrogen bonding interaction, enhancing the miscibility and affinity between PEG and NCC matrix. Herein, it is favorable to form effective heat conduction paths, which assist to improve the TC along the plane. As a result, the TC value achieved 21.05 W m^−1^ K^−1^ with 90 wt% PEG loading.

## Experimental Section

### Materials

Graphene was purchased by Ningbo Moxi Tech. Co., Ltd. (China). 3, 4, 9, 10-Perylene tetracarboxylic anhydride (PTCDA) was purchased from the Xiya Chemical Reagent Company (Chengdu, China). 6-Aminocaproic acid, imidazole, triphenylphosphine (TPP) and Calcium chloride anhydrous were all purchased from Aladdin Chemistry Co., Ltd, China. Ethanediamine (EDA), Pyridine (Py), methanol, 30% hydrochloric acid, the solvent N-methyl-2-pyrrolidone (NMP) and N, N-dimethylformamide (DMF) were analytical grade, provided by Guangdong Guanghua Sci-Tech Co., Ltd, China. Sisal fibers were bought from Guangxi Sisal Company, China. NaClO_2_, CH_2_ClCOOH, anhydrous ethanol, Na_2_SO_4_•10H_2_O, CH_3_COOH, NaOH, H_2_SO_4_ and H_2_O_2_ were purchased from Sinopharm Chemical Reagent Co., Ltd China. All other reagents and solvents were used without further purication.

### Synthesis of the perylene bismide derivatives (PBI) and polyamine-functionalized perylene bismide (PED)

**Figure 1:**
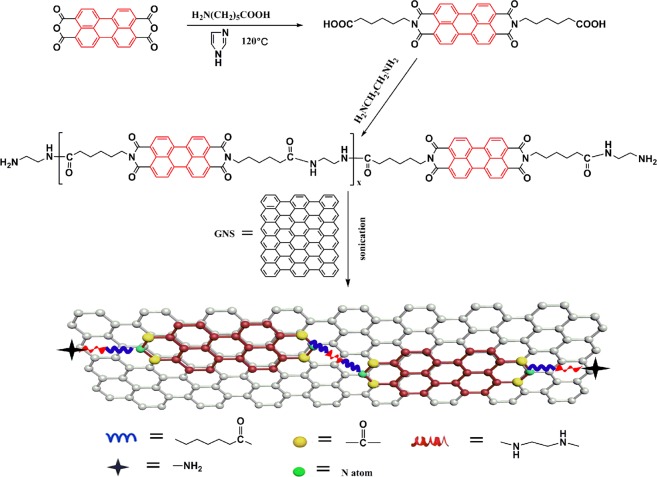
The preparation process of PEG.

The synthetic routes of PBI and PED are shown in Fig. 1. Firstly, the synthesis of PBI was performed according to a previously report^35^. 3,4,9,10-Perylene tetracarboxylic anhydride (PTCDA) (0.5 g, 1 mmol), 6-aminocaproic acid (0.4 g, 3 mmol) and imidazole (5 g) were stirred at 120 °C for 12 h under nitrogen atmosphere. Then added deionized water (50 ml) with stirring continues for another 2 h at 90 °C. Next, the dark-red solution was filtered to remove the unreacted PTCDA. The mixture was acidified with 2 M HCl aqueous solution until the pH reached a value of ~4. The resultant precipitate was filtered out several times until the filtrate was neutral and dried in a vacuum oven at 60 °C overnight. Secondly, the synthesis process of PED was as follows, the as-prepared PBI powder (0.5 g, 0.8 mmol), Calcium chloride anhydrous (0.3 g) and NMP (30 ml) were added into a round-bottom flask and purged with nitrogen gas for 15 min. EDA (0.05 ml, 0.8 mmol), TPP (0.5 ml) and Py (0.4 ml) were added into the flask under nitrogen atmosphere and stirred for 12 h at 120 °C. The mixture was poured into the 1:1 methanol/deionized water and washed at least five times. The final product is a reddish brown solid after drying in a 60 °C vacuum oven for 8 h.

### Preparation of PED functionalized graphene nanosheets (PEG)

Noncovalent functionalization of GNS by PED was carried out as follows. GNS (10 mg) was dispersed in 30 ml DMF by mixing with PED (40 mg) by sonication in water bath for 3 h to give a black homogeneous suspension. The suspension was then centrifuged at 9500 rpm for 5 min to remove the unabsorbed GNS, and the process was repeated for three times to afford the pure PEG nanocomposites. After centrifugal, the supernatant turned to golden yellow due to *π*–*π* stacking interaction. The final product is a black solid powder after drying in 80 °C vacuum oven overnight, as shown in Fig. 1.

### Preparation nanocrystalline cellulose from sisal fibers (NCC)

NCC was extracted from sisal fibers by three steps, de-waxing processing, bleaching processing, grafting processing and a series of mechanical processing. The details of the three processing were as follows. (1) De-waxing process: firstly, the sisal fibers were chopped to an approximate length of 5 mm and washed with deionized water to remove the grain shape lignin on the surface of sisal fibers. Then the sisal fibers were dried in an oven at 60 °C for later use. Secondly, the 50 g drying fibers were boiled in a mixture aqueous solution of NaOH (4 wt%) and Na_2_SO_4_•10H_2_O (4 wt%) in 500 ml autoclaves at 170 °C for 2 h. Finally, the product were filtered and washed several times and dried in oven overnight. (2) Bleaching process: the obtained de-waxing sisal fibers, 12 g NaClO_2_, 5 ml CH_3_COOH and 350 ml deionized water were added into a 500 ml three-necked flask with stirring at 80 °C for 2 h. The product were washed and filtered as above and microcrystalline sisal fibers (MSF) were obtained. (3) Grafting process: firstly, the 7 g MSF, 35 g 10 wt% NaOH and 350 ml anhydrous ethanol were added into a 500 ml three-necked round bottomed flask with stirring at 30 °C for 30 min. Then added 3.5 g monochloroacetic acid (CH_2_ClCOOH) with stirring continues for another 3 h at 70 °C. The product was collected after washing many times by centrifugal precipitation until neutral. The crude product was dispersed and soaked in 500 ml deionized water for 1 h, shearing the product into small fragments by a high-shear dispersion homogenizer at a speed of 28000 rpm for 30 min. And the transparent gel of nanocrystalline cellulose (NCC) was obtained with a solid content of 0.25 wt% for later use.

### Preparation of NCC/PEG composite papers (NPGs)

**Figure 2:**
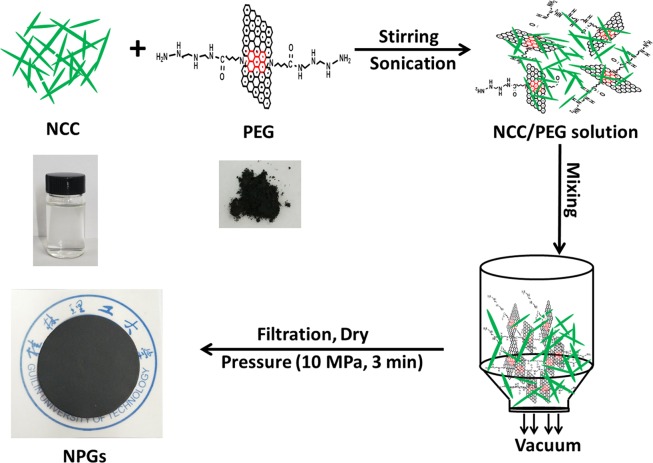
Schematic illustration of the constraction of NPGs.

At first, a NCC aqueous dispersion with a solid concentration of 0.25 wt% was mixed with PEG via fast stirring for 6 h and then sonication for 30 min. The ultrasonic power was set at 120 W to avoid damaging the PEG. Next, the dispersion was vacuum-filtered on a mixed cellulose ester membranes with a diameter of 50 mm and a pore size of 0.22 µm. The films were dried in a vacuum oven at 30 °C overnight. Finally the drying hybrid films were compressed at a tablet press (10 MPa) for 3 min. The experimental details of the process of NCC/PEG composite papers are shown in Fig. 2. The obtained hybrid films that had an NCC/PEG weight ratio of 10:0, 7:3, 5:5, 3:7 and 1:9 were denoted as NCCs, NPGs-30, NPGs-50, NPGs-70 and NPGs-90, respectively.

### Characterization

Fourier transform infrared spectra (FT-IR) were recorded on a Thermo Nexus 470 FTIR spectrometer (KBr disk). A thermal gravimetric analysis (TGA) instrument (Netzsch STA-449) was used to investigate the thermal stability up to 800 °C at a heating rate of 10 °C/min under nitrogen atmosphere. XPS measurements were recorded with an ESCALAB 250Xi instrument (Thermo Electron Corporation, US). UV-vis spectra in the wavelength range of 300–700 nm was obtained from a UV 3600 spectrophotometer (SHIMADZU Company, Japan). Fluorescence spectra were measured on a Cary Eclipse fluorescence at the excitation wavelength of 420 nm. The excitation/emission slits was set at 2.5 × 2.5 nm. Atomic force microscopy (AFM) images were taken on a multimode scanning probe microscope from Digital Instruments with Nanoscope IA controller. Field emission scanning electron microscopy (FE-SEM, JEOL JEM-6610) was used to observe the morphology of the specimens. Transmission electron microscopy (TEM) images were recorded on a JEM-2100F high-resolution transmission electron microscope at 200 kV. Samples were prepared by placing a drop of deionized water dispersion on the surface of ultrathin carbon film. The thermal diffusivity (*α*) of the nanocomposites was measured by the laser flash apparatus (LFA, NETZSCH 447, Germany) at 25 °C. The thermal conductivity was calculated as follow;

1 $$k=\alpha \times Cp\times \rho $$

Where *α* and *ρ* are the thermal diffusivity and the density of the nanocomposites, respectively. *C*_*p*_ is specific heat capacity, and measured on differential scanning calorimetry (NETZSCH DSC-204, Germany), *ρ* was calculated according to the samples’ dimension and weight. The infrared (IR) photos were captured by IR camera (Fluke, Ti400, USA).

## Results and Discussion

### Preparation and characterization of PEG

**Figure 3:**
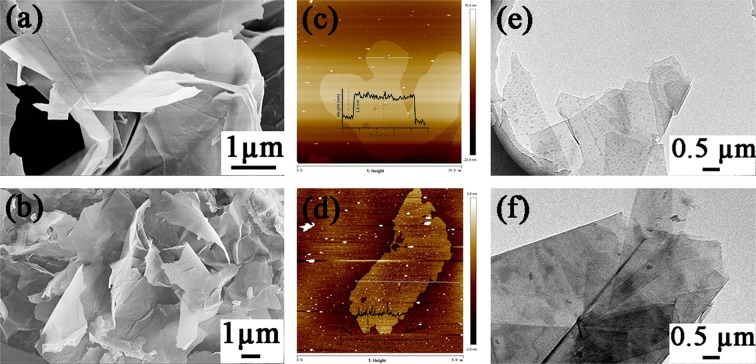
SEM, AFM and TEM images of GNS (**a**,**c**,**e**) and PEG (**b**,**d**,**f**).

PEG was prepared through the *π*–*π* stacking interaction between PED and GNS by a simple method of sonication. Compared to the covalent functionalization, noncovalent functionalization does not destroy the sp^2^ structure of graphene lattices. It can be seen from the Fig. S1(a) that the graphene demonstrates a smooth surface, while the PEG demonstrates a rough surface as shown in Fig. S1(d). Graphene tends to aggregate seriously and have much precipitate on the bottom of GNS aqueous dispersion as shown in Fig. S1(b,c), which is due to the strong interlayer van der Waals forces of graphene nanosheets. Though there are a lot of loose sediments on the PEG aqueous dispersion, all the sediments can be re-dispersed with only mild oscillation because the PED molecules were all absorbed onto the graphene sheets, as shown in Fig. S1(e,f). Figure 3 shows the SEM, AFM and TEM images of GNS and PEG. Figure 3(a,b) show the SEM images of the graphene and PEG dispersion, respectively. It can be seen from the Fig. 3(b) that PEG exhibits a scattered cluster sheets with a smooth surface. These observations suggest that the dispersion of GNS can be improved with the aid of PED. Fig. 3(c,d) show the AFM images and the high profiles of GNS and PEG, respectively. As shown in Fig. 3(c), the thickness of GNS was measured to be 1.6 nm, which indicated that the GNS has four or five layers stacked together. After PED was attached into the GNS via *π*–*π* stacking interaction, the thickness of PEG was increased to 2.8 nm (Fig. 3(d)), indicating the strong *π*–*π* stacking interactions exist between PED and GNS. Besides, the morphology of GNS and PEG were demonstrated by the TEM. As shown in Fig. 3(e), it can be seen that the GNS was several layers consistent with the AFM characterization and the edge of the GNS were rolled up. And the surface of PEG is darker than GNS and preserves the complete sheet structure as shown in Fig. 3(f), suggesting that the PED has attached onto the surface of GNS and the *π*–*π* stacking interaction does not destroy the GNS layered structure.

**Figure 4:**
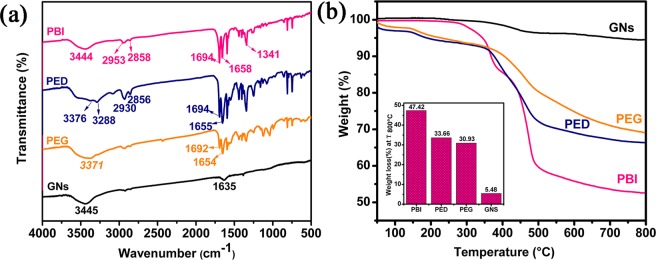
(**a**) FT-IR spectra and (**b**) TGA curves of PBI, PED, PEG and GNS.

The FT-IR spectra of PBI, PED, GNs and PEG are shown in Fig. 4(a). The FT-IR spectra of PBI exhibits a characteristic absorption band at 1694 and 1654 cm^−1^ are assigned to the carboxyl O=C-O stretching and the imide C=O stretching vibration, respectively. Additionally, the peaks at 2953 and 2858 cm^−1^ correspond to the C-H stretching vibration which comes from the 6-aminocaproic acid. The bond at 1341 cm^−1^ can be C-N stretching vibrations. Thus, these characteristic absorption bands indicate the reaction between perylene and 6-aminocaproic acid. For PED sample, the peaks at 1649 and 1655 cm^−1^ are assigned to the N-O=C stretching. And the intensity of N-C=O peak in the PED is much stronger than that in the PBI. In addition, the broad bands at 3288 and 3376 cm^−1^ are attributed to the asymmetric and symmetric vibration of –NH_2_ of PED. These changes indicate that the PBI and EDA were reacted through amide reaction. In the spectra of the GNS, the characteristic peaks at 1635 cm^−1^ is attributed to the C=C stretching vibration. Compared to the GNS, it can be found that new peaks were introduced into the PEG spectrum. These new peaks at 3371, 3283, 1692, 1654 and 1341 cm^−1^ are consistent with the peaks from spectrum of PED and become weaker due to the *π*–*π* stacking interactions^36^. These changes suggested the successful noncovalent functionalization of GNS and PED.

The GNS, PBI, PED and PEG were analyzed using TGA under nitrogen atmosphere to 800 °C at a heating rate of 10 °C/min. As shown in Fig. 4(b), the TGA curve of the PBI shows a three-step decomposition process which is attributed to the separate degradation mechanisms for the side-chains (alkyl chain segment of 6-aminocaproic acid) and the backbone (benzene ring of perylene bisimide)^37^. From the curve of PED, it can be seen that the initial decomposition temperature is degreased to 189 °C compared to the PBI (300 °C) that is ascribed to the loss of the amide chain, indicating the successful amide reaction between PBI and EDA. As shown in the Fig. 4(b), PEG has a higher decomposition temperature than that of PED owing to the good thermal stabilizing effect of GNS. TGA date indicate that PEG had a 29% weight loss at 800 °C, while the values for PED and GNS are 33.66% and 5.48%, respectively, as shown in the inset of Fig. 4(b).

**Figure 5:**
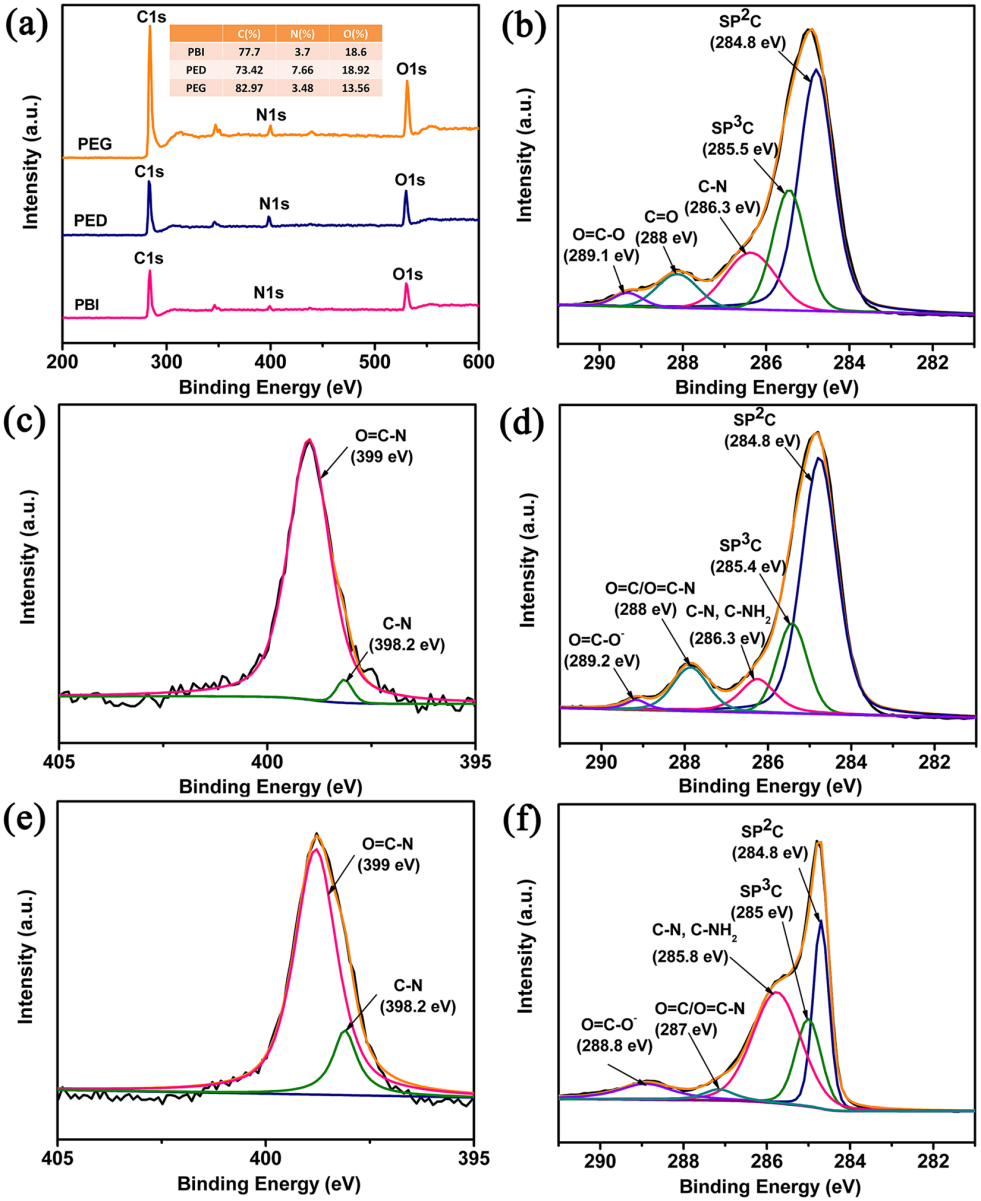
XPS general spectra of PBI, PED and PEG (**a**). C 1s core-level of PBI (**b**), PED (**d**), and PEG (**f**). N 1s core-level of PBI (**b**) and PED (**e**).

XPS characterizations, as shown in Fig. 5, were employed to further confirm that the PED was successfully synthesized and the effective noncovalent functionalization between PED and GNS. As shown in Fig. 5(a), the general spectrum of the PBI, PED and PEG all demonstrate three peaks at 284.0 eV (C 1s), 399 eV (N 1s) and 531 eV (O 1s). For PBI sample, the C 1s core-level spectrum shows five carbon components with BEs at 284.8, 285.4, 286.3, 288 and 289.1 eV, corresponding to the sp^2^ C, sp^3^ C, C-N and O=C-O groups25,26, respectively, as shown in Fig. 5(b). And for PED sample, the C 1s core-level spectrum displays five species of carbon peaks of sp^2^ C (284.8 eV), sp^3^ C (285.4 eV), C-N/C-NH2 (2856.3 eV), C=O/O=C-N (288 eV) and O=C-O^−^ (289.2 eV) groups, as shown in Fig. 5(d). The intensity peak of sp^3^ C is decreases and the intensity peak of C=O/O=C-N is increases may due to the successful amide reaction. From the surface elements composition in the inset in Fig. 5(a), the nitrogen content of PED (7.66%) is much higher than that of PED (3.7%). In addition, the enhanced intensity of the C-N peak in the N 1s core-level spectrum (Fig. 5(c,e)), the relative atomic percentage of nitrogen to carbon c (C/N) of PBI was estimated to be 21, which was higher than that of PED (9.6). These changes indicated that the lengths of EDA chains have been grafted on the PBI successfully. For PEG nanocomposite, as shown in Fig. 5(f), the C1s core-level spectrum displays five species of carbon peaks of sp^2^ C (284.8 eV), sp^3^ C (285 eV), C-N/C-NH_2_ (285.8 eV), C=O/O=C-N (287 eV) and O=C-O^−^ (289 eV) groups. The C-N/C-NH2 and C=O/O=C-N groups come from the PED and the high intensity of N 1s peak is consistent with the PED. Except for sp^2^ C, all groups have a downshift at a range of 0.5 to 1 eV compared to PED as shown in Fig. 5(d,f), which may due to a charge transfer between large aromatic molecules and the graphene sheets^26^. These changes confirmed that PED has successfully attached into the GNS.

**Figure 6:**
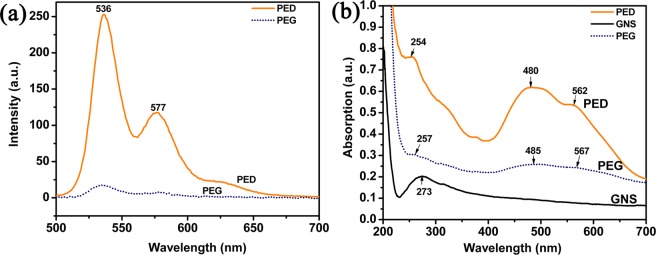
(**a**) The fluorescence emission spectrum and (**b**) UV-vis absorportion spectrum of PED and PEG in ultrapure water.

There were many reports of polycyclic aromatic precursors with graphene through noncovalent functionalization of *π*–*π* stacking interaction to disperse graphene^38–40^. The fluorescence spectroscope and the UV-vis absorbance spectroscope were commonly employed to characterize the *π*–*π* stacking interaction between graphene and polycyclic aromatic precursors, such as pyrene derivatives and porphyrin derivatives^41^. Perylene imide derivatives are a class of fluorescent substances and their fluorescence could be quenched when they are attached onto graphene nanosheets because of photoinduced electron transfer^42,43^. Figure 6(a) shows the fluorescence spectra of PED and PEG. It can be seen that PED displays two fluorescence peaks at 536 and 577 nm which are characteristic of the emission of perylene group, quenched after being attached onto graphene. A UV-vis absorption spectroscopy was also carried out to confirm the successful *π*–*π* stacking interaction between PED and graphene. As shown in Fig. 6(b), the peaks at 485 and 567 nm are attributed to the perylene groups and have a slight red shift in the PEG spectrum. Both the fluorescence spectroscope and the UV-vis absorbance spectroscope verified the successful *π*–*π* stacking interaction between PED and graphene.

### Morphology

**Figure 7:**
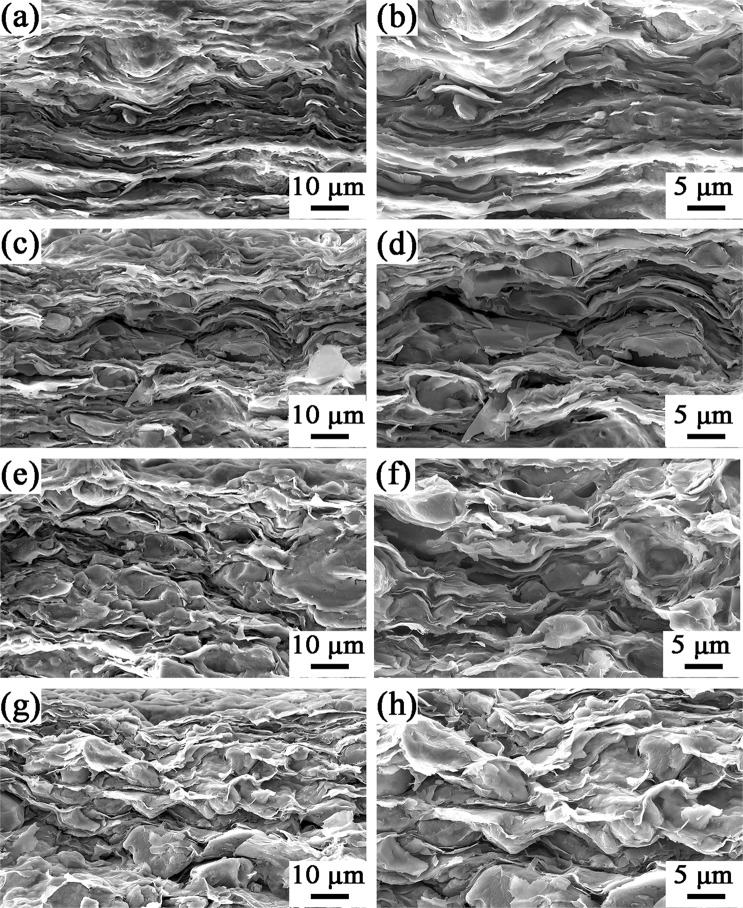
The cross-sectional SEM images of (**a**,**b**) NPGs-30, (**c**,**d**) NPGs-50, (**e**,**f**) NPGs-70 and (**g**,**h**) NPGs-90.

The digital photograph of high-transparent of NCC aqueous solution is displayed in Fig. S2(b). Besides, the TEM was employed to demonstrate the morphology of NCC and NCC/PEG dispersion, as shown in Fig. S2(a,d). Its average length and diameter of NCC were about 0.6 µm and less than 10 nm, respectively. What’s more, it can be seen that there is much NCC absorbed onto the surface of PEG as shown in Fig. S2(d), indicating that NCC and PEG have a good compatibility. As shown in Fig. S2(c), NCCs is very transparent and have a good flexible property, so even the NPGs-90 demonstrates excellent flexibility shown in Fig. S2(f). The NCC/PEG uniform dispersion was prepared and stayed without precipitation except for NCC/PEG-90 at least 24 h, as shown in Fig. S3. The formation mechanism of the homogeneous dispersion of NCC/PEG is attributed to such two reasons. On the one hand, the preparation of NCC has introduced many carboxyl groups which produced a repulsive force to form a stable NCC dispersion^44^. On the other hand, the noncovalent functionalization of graphene with PED which not only improves the dispersion of GNS, but also introduces many –NH_2_ groups that can form the hydrogen bonding interaction, improving the compatibility of PEG and NCC. The cross-section morphology of the NPGs was carried out by SEM, as shown in Fig. 7. It can be seen that the layered structure is obvious and cellulose molecular chains tend to lie along the in-plane directions. This is attributed to stable dispersion of NCC/PEG. Besides, the one-dimensional NCC was well combined with two-dimensional graphene and organized well under the vacuum-assisted filtration process, forming a high-orientation layered structure. What’s more, the mechanical pressure also made the layered structure more compact reported in a recent article^30^. When PEG loading was over 70 wt%, the layered structure was not obvious which is due to the excessive PEG is not evenly dispersed in the NCC matrix.

### Thermal conductivity

**Figure 8:**
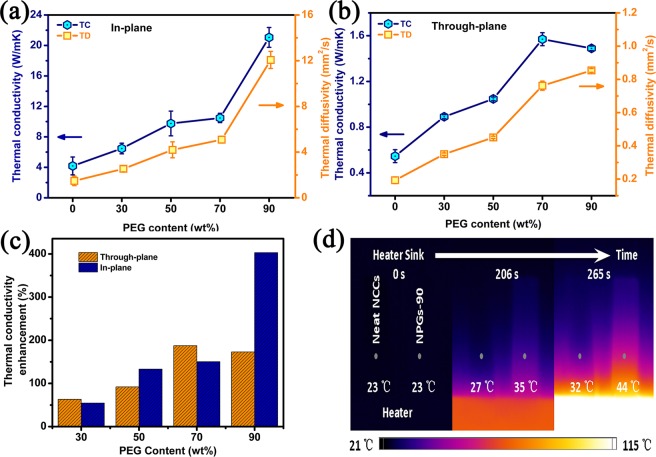
(**a**) In-plane and (**b**) Through-plane thermal conductivity and thermal diffusivity (**c**) Thermal conductivity enhancement and (**d**) IR thermal images of neat NCCs and NPGs-90 at different heating times.

Figure 8(a,b) show the in-plane and through-plane thermal conductivities (TC) and thermal diffusivity (*α*) of the NPGs, respectively. The neat NCC film has a high in-plane TC of 4.18 W m^−1^ K^−1^ at 25 °C, which is higher than other polymer matrix that have low TC values of 0.1–0.5 W m^−1^ K^−1^ ^45^. According to a previous report, the in-plane TC of single nanocrystalline cellulose varies from 0.72 to 5.7 W m^−1^ K^−1^ ^46^. This can be ascribed to the degree of NCCs alignment and the types of cellulose^47^. For the in-plane direction, the TC and *α* value increased with the increase of PEG loading and reached 21.05 W m^−1^ K^− 1^ and 12.07 mm^2^/s with a 90 wt% PEG, respectively. Furthermore, the NPGs-90 has a 403% thermal conductivity enhancement (TCE) relative to the neat NCC films (4.18 W m^−1^ K^− 1^) in the plane as shown in Fig. 8(c). After a careful comparison, we find that the thermal conductivity of our results is comparable of much higher than other nanocellulose-based composites, as shown in Table 1. The good thermal transport properties of NPGs are attributed to the strong hydrogen-bonding interaction between nanocrystalline cellulose and graphene and the well orientation of graphene. It can be seen from the Fig. 8(c) that the TC significantly increase from 70 to 90 wt%. This can be explained by the TGA analysis (Fig. S4), it was figured out that PEG contains about 10 wt% GNS and 90 wt% PED. Therefore, GNS only accounts for 10% of different amounts of PEG. When the PEG content are 0, 30, 50, and 70 wt%, the TC were increased slight may due to the little contact between graphene, thus no good thermal conduction path is formed. But when the PEG content is 90 wt%, the more contact area between graphene is formed and the TC increases sharply. The thermal conduction shows percolation threshold phenomenon in the NPGs, as previously reported^48^. For the through-plane direction, the TC reaches only 1.57 W m^−1^ K^−1^ at the loading of 70 wt% of PEG, which is attributed to highly aligned PEG network throughout the NCC matrix, demonstrating a high anisotropy values (presented as the in-plane TC divided through-plane TC), as shown in Table S1. In the through-plane, it can be seen from the Fig. 7 that the NPGs-70 have a clearer stratification than NPGs-90 due to the poor dispersion of NCC/PED-90, thus blocked the through-plane’s heat conduction paths. So there is a slightly decrease of TC from 70 to 90 wt%. To further investigate the potential application of NPGs in electronic devices, an IR imaging spectrometer was employed to characterize the thermal transfer performance of neat NCCs and NPGs, as shown in Fig. 8(d). Neat NCC and 90 wt% NPGs composite strips acted as the heat spreader. The size of each strip was 20.0 (length) × 5.0 (width) × 0.08 mm (thickness). One end of the strip was connected to a heater. As shown in Fig. 8(d), IR images demonstrate the temperature increase of neat NCCs and NPGs composites from one side to another, respectively, as heating time goes. The temperature of the samples at the same point of the heater was compared. Before heating, the whole device stayed at 23 °C. The NPGs was heater than neat NCCs. When the heater reached 115 °C, the temperature of the NPGs (44 °C) is 12 °C higher than NCCs (32 °C). This consequence further proved that PEG-reinforced NCCs have a better efficiency in the real case, which is in accordance with the higher In-plane thermal conductivity of NPGs.

**Table 1:** In-plane TC of Nanocellulose-based composites and graphene-filled composites

Composites	TCE (%)	Filler content (wt%)	In-plane TC (W m^−1^ K^−1^)	References and year
NFC/ reduced graphene oxide	550	30	6.618	2015^50^
NFC/ graphene sheets	410.7	10	5.73	2017^31^
NFC/ Boron Nitride Nanotubes	1375.17	25	21.39	2017^51^
NFC/ Nanodiamond	775.2	0.5	9.820	2017^16^
NFC/ functionalized graphene	247.5	6	9.0	2017^52^
Epoxy/graphene sheet	2258.8	10.1	4.01	2014^53^
Polystyrene/graphene	66	10	0.244	2015^54^
PA6/ graphene nanoribbons	165	0.5	4.85	2015^55^
PA6/3D graphene	300	2.0	0.847	2016^56^
PI/reduced graphene oxide	52.9	2.0	0.26	2016^57^
NFC/functionlized grapheme (NPGs-90)	403	10	21.05	This work

NFC = nanofibrillated cellulose, PA6 = polyamide-6, PI = polyimide.

**Figure 9:**
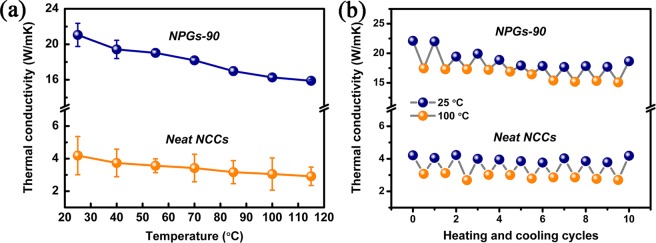
(**a**) Temperature-dependent thermal conductivity for neat NCCs and NPGs-90. (**b**) Thermal conductivity of NCCs and NPGs-90 over 10 heating/cooling cycles.

The temperature dependence of the in-plane thermal conductivity was studied. As shown in Fig. 9(a), the thermal conductivity of the NPGs-90 decreases with increasing temperature, which is consistent with the trend of neat NCCs. The decreased thermal conductivity may be attributed to the Umklapp phonon scattering processes for the crystalline phase in NCCs^49^. Figure 9(b) shows ten heating/cooling cycles alternating between 25 and 100 °C. The thermal conductivities of NCCs and NPGs almost maintain the original thermal conductivity and have a slight thermal conductivity decrease after ten heating/cooling cycles, indicating stable capability of heat conduction in this temperature range.

## Conclusions

In summary, we fabricated NPGs of high thermal conductivity by vacuum-assisted self-assembly method. With PED, high concentration of aqueous PEG dispersion up to 0.5 mg ml^−1^ was obtained at the weight ratio of PED to graphene to 4:1. Meanwhile, it was found that PEG and NCC matrix can form stable dispersions due to the good compatibility of graphene-NCC through the hydrogen bonding interaction, which contributed to the PEG well-aligned in a NCC matrix. It is favorable to form effective heat conduction paths, which assist to improve the in-plane TC. As a result, the in-plane TC of NPGs-90 reached 21.05 W m^−1^ K^−1^ with 90 wt% PEG loading, which was 403% enhancement relative to the pure cellulose paper.

## Supplementary information

Tailoring Thermal Transport Properties of Graphene Paper by Structural Engineering
