# Supplementary material for: Tailoring Thermal Transport Properties of Graphene Paper by Structural Engineering
Source: Sci Rep. 2019 Mar 14;9:4549. doi: 10.1038/s41598-018-38106-0 (PMC6418276; doi:10.1038/s41598-018-38106-0)
Supplement: Supplementary file 1 — Tailoring Thermal Transport Properties of Graphene Paper by Structural Engineering [file 41598_2018_38106_MOESM1_ESM.docx]

**Supporting Information**

**Tailoring Thermal Transport Properties of Graphene Paper by Structural Engineering**

Li Ren^1^, Mengjie Wang^2^, Shaorong Lu^1,*^, Lulu Pan^1^, Zhongqiang Xiong^1^, Zuocai Zhang^1^, Qingyuan Peng^1^_,_ Yuqi Li^1^, and Jinhong Yu ^2,*^

^1^Key Laboratory of New Processing Technology for Nonferrous Metals and Materials, Ministry of Education, School of Material Science and Engineering, Guilin University of Technology, Guilin, 541004, China

^2^Key Laboratory of Marine Materials and Relater Technologies, Zhejiang Key Laboratory of Marine Materials and Protective Technologies, Ningbo Institute of Materials Technology and Engineering, Chinese Academy of Sciences, Ningbo 315201, China

*Corresponding authors E-mails: [**lushaor@163.com**](mailto:lushaor@163.com) **(S. R. Lu.);** [**yujinhong@nimte.ac.cn (J**](mailto:yujinhong@nimte.ac.cn%20(J)**. H. Yu).**

**SUPPLEMENT**

**
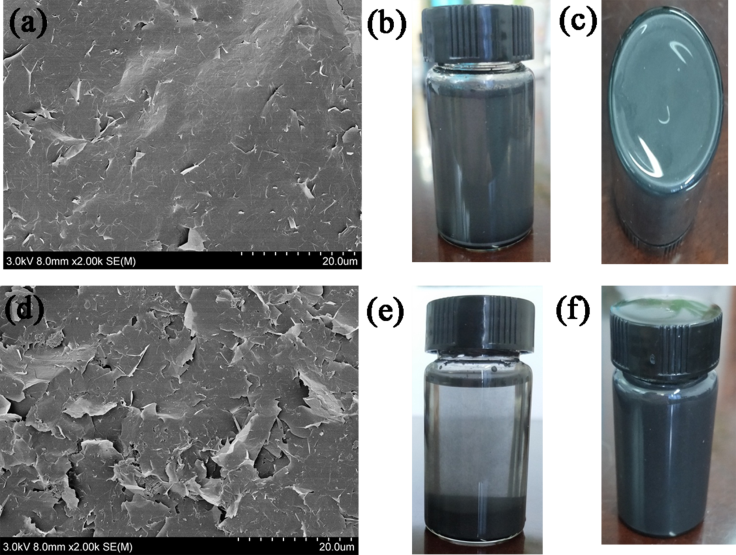
**

**Figure S1.** SEM images of GNS and PEG (a, d). Digital images of GNS and PEG aqueous dispersion (0.5 mg/ml) (b, e). The right images show the dispersion with much precipitate on the bottom of GNS aqueous dispersion (c) and the redispersed PEG aqueous dispersion with only mild oscillation (f).

**
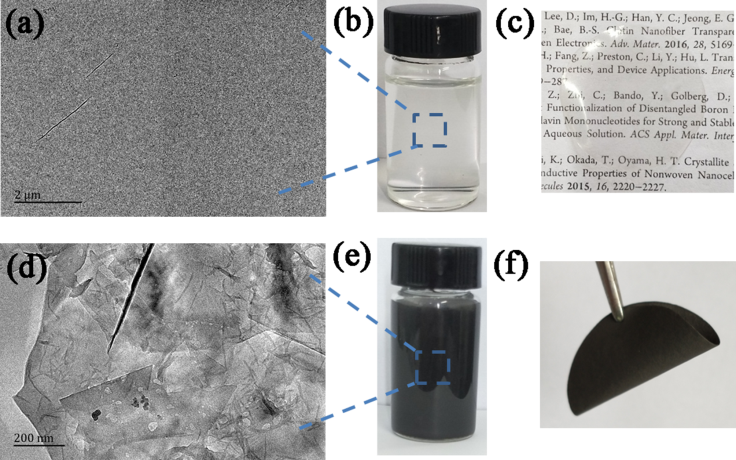
**

**Figure S2.** TEM images of NCC and PEG aqueous solution (a, d). Digital images of NCC and PEG aqueous solution (b, e) and digital images of NCCs and NPGs-90 (c, f).


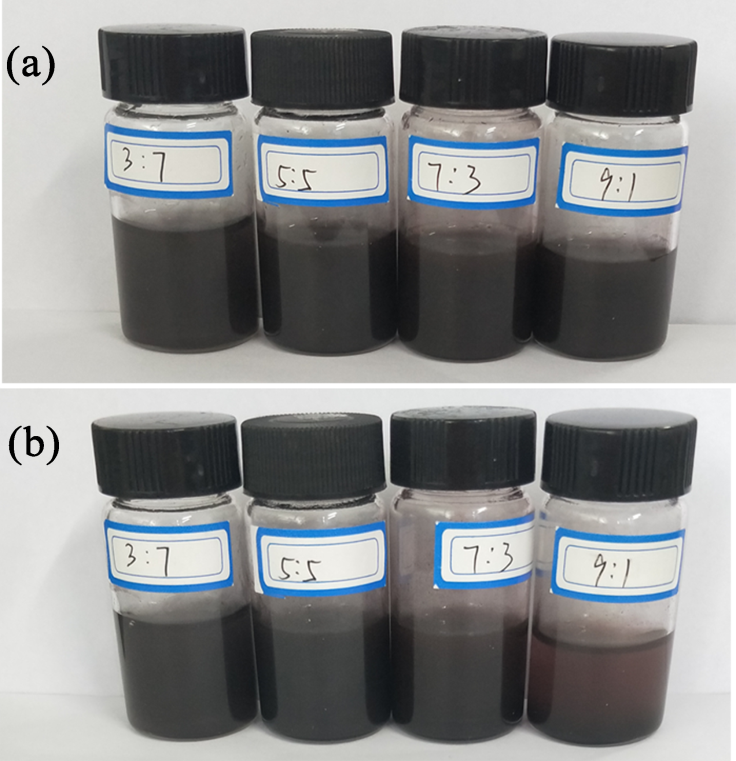


**Figure S3.** (a) Digtal images of the NCC/PEG-30, NCC/PEG-50, NCC/PEG-70 and NCC/PEG-90 dispersion after stirring for 6h and sonication for 30min. (b) Digtal images of the NCC/PEG-30, NCC/PEG-50, NCC/PEG-70 and NCC/PEG-90 dispersion after standing for 24 h.





**Figure S4.** TGA curves of PED, PEG and GNS.

It can be seen from the inset of **Figure S4** that PEG had a 30.93 % weight loss at 800 °C, while the values for PED and GNS are 33.66 % and 5.48 %, respectively. Thus, it was figured out according to the following equation that PEG contained about 10 wt% GNS and 90 wt% PED. $33.66x+5.48y=30.93$ (1)

$x+y=1$ (2)

*x* and *y* are weight loss percentage of PED and GNS, respectively.

**Table S1** Anisotropy values of the NPGs.

| **Sample** | **NPGs-30** | **NPGs-50** | **NPGs-70** | **NPGs-90** |
| --- | --- | --- | --- | --- |
| Anisotropy values | 7.26 | 9.31 | 6.68 | 14.13 |
